# Development of immunocompetent models for primary and metastatic ER+ breast cancer

**DOI:** 10.1002/ame2.70210

**Published:** 2026-05-12

**Authors:** Devon M. Bull, Jody Hazlett, Emily Schulpen, Sophie Tunnicliffe, Alexander D. McLellan, Anita K. Dunbier

**Affiliations:** ^1^ Department of Biochemistry University of Otago Dunedin New Zealand; ^2^ Department of Pathology and Biomedical Sciences University of Otago Christchurch New Zealand; ^3^ Department of Microbiology and Immunology University of Otago Dunedin New Zealand

**Keywords:** breast cancer, estrogen receptor positive, immunocompetent model, metastasis

## Abstract

**Background:**

The development of effective therapeutic strategies for late‐stage estrogen receptor‐positive breast cancer (ER+) is limited by the scarcity of biologically relevant models. More recently, immunotherapies emerged as promising candidates for breast cancer treatment, however, the absence of immunocompetent models of ER+ breast cancer metastasis continues to hinder the assessment of these theraputic interventions.

**Methods:**

To address this, we utilized the 129S6/SvEv mouse strain and syngeneic SSM3 cells in the assessment and development of ER+ metastasis models. As part of this study, the mammary intraductal (MIND) primary tumor model was established in the same background. In addition, a novel luciferase system was evaluated for potential use in metastasis tracking.

**Results:**

Luciferase‐expressing SSM3 cells enabled longitudinal in vivo imaging to track tumor growth. Histological analysis confirmed metastatic spread and tumor origin. Antares2, a novel luciferase reporter, showed high in vitro sensitivity but reduced in vivo performance. The study showed that systemic delivery of SSM3 cells with oestradiol supplementation can support metastatic tumor establishment and that MIND injections led to reliable, invasive tumor growth.

**Conclusions:**

These findings highlight the potential and limitations of the 129S6/SvEv model as a syngeneic, immunocompetent system for studying ER+ breast cancer metastasis. Reporter expression may affect immunogenicity or cell fitness. Further refinement of these models will enable investigation of immune‐modulatory therapies in ER+ metastatic breast cancer.

## Graphical abstract

This study explores syngeneic, immunocompetent models of ER+ breast cancer in 129S6/SvEv mice, using trackable tumor cells to model both primary tumors and metastasis. The models show promise, while also revealing limitations for studying advanced disease and immune interactions.

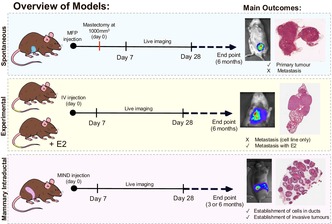

## 1 INTRODUCTION

Breast cancer is the most prevalent cancer among women globally and is the second highest cause of cancer‐related deaths.^1^, ^2^ Estrogen receptor positive (ER+) is the most common subtype of breast cancer, accounting for approximately 70%–80% of all breast cancer cases. ER+ breast cancer is primarily driven by estrogen signaling, which activates downstream gene expression and promotes tumor growth and progression.^2^, ^3^, ^4^ The current standard of care includes anti‐hormone therapies, however, therapy resistance remains a significant clinical challenge. Consequently, the development of new therapeutic approaches, including immunotherapies, is critical for improving patient outcomes and broadening available treatment options.^5^, ^6^ Metastatic breast cancer (mBC) remains incurable despite current treatments, such as cytotoxic therapies, surgery, radiation therapy, and targeted therapies.^7^ Mouse models are crucial for advancing our understanding of breast cancer and developing targeted therapies.^8^ However, it is challenging to capture the entire spectrum of factors involved in disease progression, such as the tumor microenvironment (TME), immune system involvement, or therapy resistance. Cell line‐derived xenografts (CDX) and patient‐derived xenografts (PDX) have been widely employed to model both primary and metastatic breast cancer.^9^ While these models have been valuable for studying tumor biology and therapeutic responses, their utility in investigating immune interactions in the context of ER+ breast cancer is limited. A significant challenge in advancing more effective treatments lies in the absence of models that accurately recapitulate the biological processes involved in metastasis. As the use of immune modulatory therapies are becoming of increasing promise, the development of an immunocompetent preclinical model will allow for these to be investigated in the context of ER+ mBC.^10^, ^11^, ^12^. Here, we aimed to develop an immunocompetent ER+ breast cancer metastasis model which can be utilized for future immune system and therapy related studies.

An immunocompetent model of ER+ breast cancer that closely resembles luminal‐like breast cancer, was produced through longitudinal studies of *Stat1* ^−/−^ 129S6/SvEv mice.^13^ The SSM3 cell line was derived from spontaneous mammary tumors produced by this model, and is one of few cell lines that is reliably estrogen responsive and overlaps with human ER+ breast cancer at the gene expression level.^13^ SSM3 cells in the 129S6/SvEv background have been used in a range of ER+ breast cancer studies, including primary tumor models involving immunotherapies, determining the effect of tumor mutational burden, and metastasis studies involving direct injection into bone.^14^, ^15^, ^16^ These tumors are responsive to estrogen and the immune system is modulated through anti‐hormone therapies.^15^, ^17^

Spontaneous and experimental models of breast cancer are commonly utilized for metastasis progression and therapy research.^18^, ^19^ While these models have been essential in disease studies and therapeutic discovery, both are limited by their level of biological relevance due to their circumvention of major milestones in breast cancer progression.^19^, ^20^ More recently, the mammary intraductal (MIND) model of breast cancer was developed, which more closely models events in luminal breast cancer.^21^, ^22^ Initially used as a model for ductal carcinoma in situ, the MIND model has now been utilized in both CDX and PDX studies of invasive and metastatic breast cancer.^21^, ^22^ In terms of models for ER+ breast cancer metastasis these are limited to an immunocompromised context, leaving a gap for more relevant models that can also encompass immune system involvement.

In addition to model development, this study aims to assess the novel Antares2 reporter system for tracking metastatic disease progression. Antares2 utilizes bioluminescent resonance energy (BRET) through a *teLuc* luciferase, cyOFP1 fluorescent protein fusion.^23^, ^24^ This reporter system is an optimized red‐shifted bioluminescent reporter, that was developed to overcome the limitations of imaging blue wavelengths in vivo.^23^, ^25^, ^26^ It has been shown to be superior to Firefly luciferase in vitro, and has also been reported to work well in vivo, yielding consistently bright signal.^23^, ^24^

## 2 METHODS

### 2.1 Cell lines and culture conditions

The SSM3 cell line, derived from spontaneous mammary tumors of *Stat1^−/−^* deficient 129S6/SvEv mice were cultured as previously described.^13^ Transfected cells were selected with 3 μg/mL puromycin. Conditioned media was collected and filter sterilized through a 0.22 μm syringe filter, for use during clonal expansion following transfections.

### 2.2 Cloning and transfections

The Antares2 and Luc2 inserts were cloned into pSBbi‐GP (addgene #60511) and pSBbi‐RP (addgene #60513) vectors via asymmetric SfiI site ligation, to generate pSBbi‐Antares2 and pSBbi‐Luc2 plasmids, respectively. Vectors were co‐transfected with the pCMV(cat)T7‐SB100 transposase vector (addgene #34879). To generate the SSM3‐A2 and SSM3‐Fl cell lines, SSM3 cells were transfected via electroporation (Neon transfection system, Thermo Fisher Scientific) using the manufacturer's MCF7 cell protocol. Fluorescent expression was then monitored every 24 h. Puromycin was added at 48 h post‐transfection for selection. Conditioned media was used to maintain healthy cultures. Cells were stable after 14 days of antibiotic selection.

### 2.3 Luciferin substrate preparation

D‐Luciferin potassium salt (GoldBio) was prepared for in vitro and in vivo use according to manufacturer's instructions. Injections were delivered intraperitoneally (i.p.) as detailed by the manufacturer.

For in vitro experiments diphenylterazine (DTZ) was reconstituted in DTZ buffer (5 mmol/L L‐ascorbic acid, 50% 1,2‐propanediol (v/v) and 50% ethanol (v/v)) resulting in a 1 mmol/L stock. DTZ at 30 μmol/L final was used for in plate assays. For in vivo experiments, DTZ was reconstituted at 2 mmol/L in in vivo DTZ buffer (25% (w/v) hydroxypropyl‐β‐cyclodextrin and 20% (v/v) propylene glycol in 1X PBS). Fluorofurimazine, FFz (Promega), was used in in vivo experiments only. FFz was reconstituted and delivered to mice as according to the manufacturer's instructions.

### 2.4 In vitro characterization

Proliferation assays were used to assess whether transfections affected SSM3 cell growth. Transfected cells were seeded in 96‐well plates (1 x 10^5^ cells/mL); at each time point, plates were fixed with 1.5 mg/mL saponin and 0.5% PFA containing 2 μg/mL Hoechst and imaged on a BioTek Cytation 5 (Agilent Technologies, Inc.). Protein was isolated from SSM3, SSM3‐A2, and SSM3‐Fl at ≥50% confluency using ice‐cold RIPA buffer and quantified by BCA. Lysates (50 μg) were combined with DTZ or LARII substrate (Thermo Fisher Scientific Firefly dual luciferase kit) and imaged on a CLARIOstar plate reader (BMG LABTECH); PBS‐only controls were included for autoluminescence. Whole‐cell flux assays quantified luciferase activity in SSM3‐A2 and SSM3‐Fl cells using the IVIS X5 system (Perkin Elmer). Cells were plated in black 96‐well plates, 30 μmol/L DTZ or 150 μg/mL D‐luciferin was added and plates were incubated for 10 min before imaging with an open emission filter (1–5 s exposure). Relative flux was measured using Living Image software and exported for analysis.

### 2.5 In vivo experiments and ethics statement

All experiments with animals were reviewed and approved by the Animal Welfare and Ethics Committee of the University of Otago and were conducted under the approved animal use protocols (AUP‐20‐125, AUP‐22‐116 and AUP‐23‐14) and performed in accordance with the University of Otago guidelines for animal research. All personnel involved were approved by the Animal Welfare Office of the University of Otago.

Female 129S6/SvEv mice (Taconic), 8–12 weeks, ~20 g, were housed in pathogen‐free rooms under 12 h light cycles with ad libitum irradiated diet (Teklad Global 18% Protein, Inotiv, USA) and water. Mice were monitored and weighed twice weekly. Anesthesia was via isoflurane (3%–4% induction, 1.5%–2% maintenance).Euthanasia was by cervical dislocation under anesthesia. Each model included one SSM3 imaging control (luciferase negative) to confirm luciferase activity was only present in SSM3‐A2 and SSM3‐Fl groups.

### 2.6 Spontaneous metastasis model

Suspensions of 1 × 10^6^ SSM3 cells in 30–50 μL ice cold 1X PBS were prepared and injected into the 4th inguinal mammary fat pad (mfp) of 129S6/SvEv mice (27G needle) under 1.5%–2% isoflurane.^27^ Hair was removed and skin sterilized prior to injection. Tumors were monitored with calipers until 1000 mm^3^, at which point a partial mastectomy was performed, or earlier if ulceration was suspected. Luciferase imaging confirmed primary tumor activity before surgery, and mice were imaged periodically for 6 months thereafter. At endpoint or early tumor regrowth, mice were euthanised,and organs were imaged and fixed for histology.

### 2.7 Experimental (tail vein) metastasis model

The SSM3‐Fl line was used for all experimental metastasis model groups: mice received 1 × 10^6^ cells, 2 × 10^6^ cells, or 2 × 10^6^ cells plus oestradiol (E2). E2 mice had drinking water supplemented with 10 μg/mL 17β‐oestradiol, replenished twice weekly. The parental SSM3 line served as a luciferase negative control during imaging. An E2‐only control was include to monitor effects of hormone supplementation. Cells were suspended as 100–150 μL injections in ice cold 1X PBS and stored on ice. Suspensions we injected via the lateral tail vein under isoflurane anesthesia, with warming to promote vasodilation. To confirm cell viability, cells were passed through a 29G needle, trypan blue–stained and tested for adhesion. Mice were imaged for luciferase activity at 1 week, monthly, and at 6 months before euthanasia and tissue collection.

### 2.8 Mammary intraductal injections

SSM3 or SSM3‐Fl cells (5 × 10^4^ or 1 × 10^5^ in 10 μL ice‐cold PBS) were injected into the 4th mammary duct of mice under isoflurane. Trypan blue (0.4%) was used to guide needle placement. Cells were delivered via a blunt 30G Hamilton syringe. Lignocaine/prilocaine cream (25 mg/g) and paracetamol (1 mg/mL in drinking water for 4 days) provided analgesia. Mice were monitored twice weekly for 3–6 months, including bioluminescent imaging. At endpoint, mice were euthanised, and mammary glands and associated tissues were collected for histology.

### 2.9 In vivo imaging

Mice were anesthetised with isoflurane, luciferin was delivered i.p. (29G), and hair was removed at imaging sites. Images were acquired using an open emission filter, binning factor 8, and 0.2 s–5 min exposures. Strong primary signals were masked with black cotton to prevent diffusion. Images were analyzed with Living Image software, with minimum counts set to 600. ROIs were placed manually, flux recorded, and time courses processed together. Data was exported to Prism 9 for analysis.

### 2.10 Histology preparation and analysis

Fixed and embedded tissues were serial sectioned (4–6 μm sections) then stained with hematoxylin and eosin (H&E). Matched adjacent sections were taken for immunofluorescent staining. Immunofluorescence (IF) was used to confirm tumors were derived from SSM3‐Fl cells by staining for tdTomato expression (tdTomato/RFP primary antibody, 1:100 dilution, pre‐ absorbed polyclonal rabbit antibody, Thermo Fisher Scientific #600‐401‐379 and Alexa Fluor 594 secondary antibody, 1:500 dilution, chicken anti‐rabbit, cross‐absorbed, Thermo Fisher Scientific #A‐11005) and cured with ProLong Gold Antifade Mounting medium with DAPI (Thermo Fisher Scientific) prior to fluorescent imaging. Fluorescent filters used for imaging were as follows, GFP (for cyOFP1 and GFP) = 460–500 nm, TxRed (for tdTomato and Alexa Fluor 594) = 540–580 nm, DAPI (for DAPI) = 340–380 nm.

### 2.11 Statistics

For in vitro assays, three independent experiments were performed. Welch's two‐tailed *t*‐test were used when comparing two groups. For in vivo analysis and comparisons between multiple groups, two‐way ANOVA was used. Statistics were calculated using GraphPad Prism 9, version 9.5.1 (San Diego, CA, USA).

## 3 RESULTS

### 3.1 Development and characterization of bioluminescent SSM3 cell lines for tracking disease progression

**FIGURE 1:**
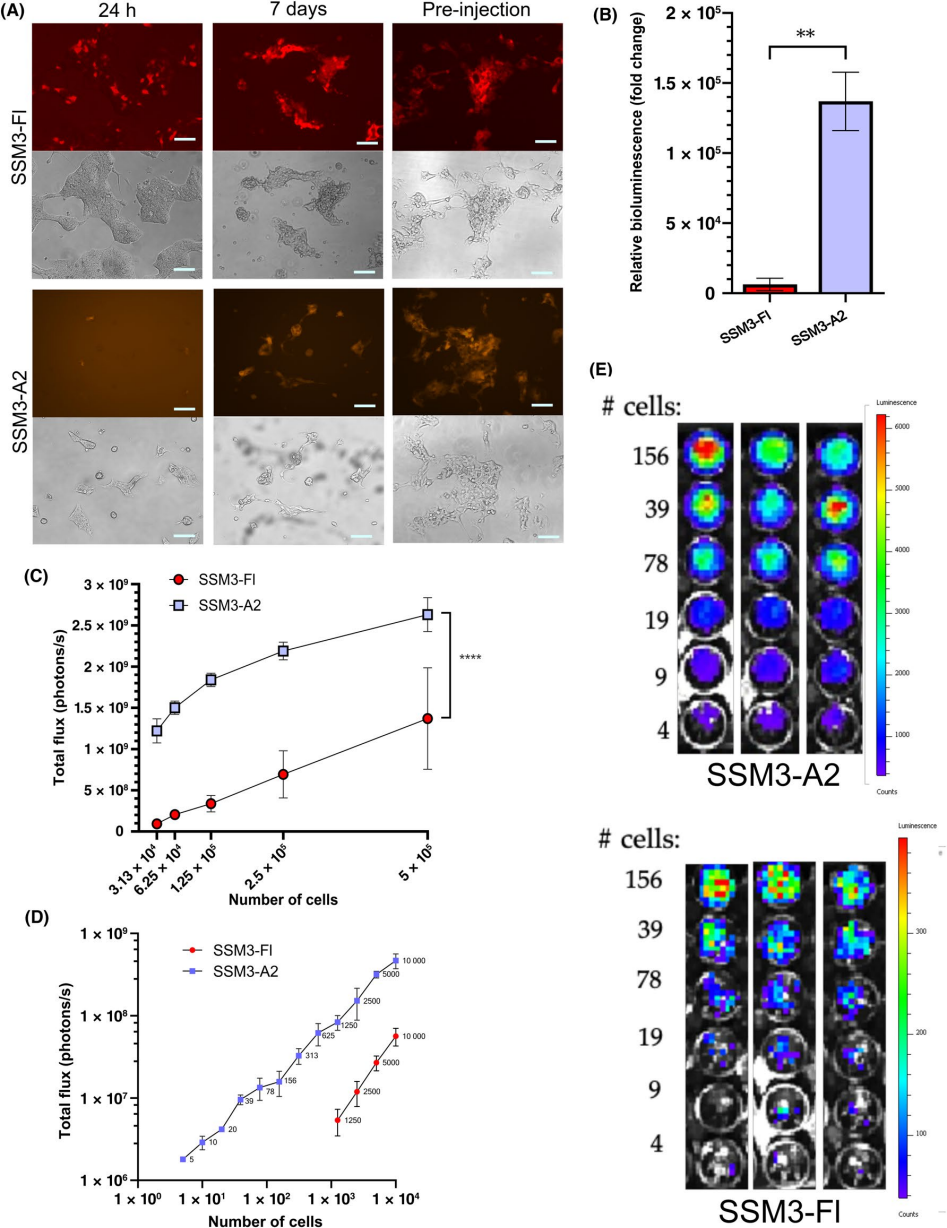
Characterization of the SSM3‐A2 and SSM3‐Fl cell lines in vitro. (A) Representative fluorescent images of SSM3‐Fl and SSM3‐A2 cells at Day 7 and 14 of puromycin selection. SSM3‐Fl and SSM3‐A2 cells were imaged using TxRed or GFP excitation filters, respectively (upper panels) and brightfield (lower panels). Scale bar = 100 μm. Luciferase activity was measured following the addition of D‐luciferin (SSM3‐Fl, red) and DTZ (SSM3‐A2, blue) substrates (B–E). (B) Luciferase expression from 50 μg protein lysates was measured on the CLARIOstar plate reader and average relative bioluminescence was plotted as the fold change of the control (parental SSM3 cells), error bars represent standard deviation. Welch's two tailed *t*‐test *p* ≤ 0.01 (**), *n* = 3. (C) Flux assay of live cells used to compare relative bioluminescence and determine total flux per individual cell. Welch's two‐tailed *t*‐test *p* ≤ 0.0001 (****), *n* = 3, error bars represent standard deviation. (D) Flux assay of live cells to determine the lower detection limit of each cell line following addition of 30 μM DTZ or 150 μg/mL D‐luciferin substrate. Average total flux (photons/s) of SSM3‐Fl and SSM3‐A2 containing wells that produced signal above background levels (<600 counts) was plotted, error bars represent standard deviation. (E) Bioluminescent images of plated cells from lower detection limit flux assay. Scale bar represented as counts. Maximum counts from SSM3‐Fl = 395.

To generate luciferase expressing cell lines the 129S6/SvEv (STAT1 KO)‐derived cell line SSM3^13^ was transfected with plasmids containing either Firefly (Luc2) luciferase or the novel reporter, Antares2^23^ (Figure S1). Positive fluorescent expression was used to confirm vector expression (Figure 1A). Proliferation assays performed with the SSM3‐A2, and SSM3‐Fl cell lines revealed that stable transfection had no detectable impact on cell proliferation (Figure S2).

To characterize the luciferase activity of SSM3‐Fl and SSM3‐A2, protein lysate and whole cell assays were performed. The average relative bioluminescence from SSM3‐A2 cell lysates was approximately ~21 fold greater than that of the SSM3‐Fl cells lysates (*p* = 0.0064) (Figure 1B). In addition, whole cell assays using the IVIS X5 bioimager revealed that SSM3‐A2 cells had approximately two times higher measures of total flux compared to the SSM3‐Fl cells (Figure 1C). Whole cell assays using a lower cell number range (1 × 10^4^ to 4 cells per well) showed the minimum number of cells detectable was ~4 cells per well for SSM3‐A2 compared to 1250 cells per well for SSM3‐Fl (Figure 1D,E, Figure S3).

### 3.2 Evaluation and characterization of cells in vivo

To model spontaneous metastasis in 129S6/SvEv mice, SSM3‐Fl and SSM3‐A2 cell lines were injected into the mammary fat pad resulting in the growth of primary tumors.^27^ Mice receiving SSM3‐Fl cells, had a 57% tumor establishment rate and mice receiving SSM3‐A2 cells, had an establishment rate of 46%, with palpable tumors forming between 3 and 6 weeks, and 2–10 weeks, respectively (Table 1).

**TABLE 1:** Summarized data from 129S6/SvEv metastasis models with SSM3 cell lines.

	Group	Experimental parameters						
		Number of mice included	Percentage of established primary tumors (*n*)	Incidence of tumor regrowth (%)	Incidence of tumor ulceration (%)^a^	Luciferase signal at 1 week^b^ (%)	Mice with suspected metastasis^c^	Confirmed metastasis (y/n)
Spontaneous (mfp)	SSM3‐Fl	7	57 (*n* = 4)	28 (*n* = 2)	28 (*n* = 2 after)	n/a	0	No
	SSM3‐A2	11	46 (*n* = 5)	27 (*n* = 3)	36 (*n* = 2 before, 2 after)	n/a	0	No
	SSM3 imaging control	2	100 (*n* = 2)	n/a	n/a	n/a	n/a	n/a
Experimental (IV)	1 × 10^6^ cells	3	n/a	n/a	n/a	100	0	No
	2 × 10^6^ cells	3^d^	n/a	n/a	n/a	100	0	No
	2 × 10^6^ cells + E2	3^d^	n/a	n/a	n/a	100	33 (*n* = 1)	Yes
	E2 control	1	n/a	n/a	n/a	n/a	n/a	n/a

^a^ Tumor ulceration occurrence before tumor removal surgery or in tumors that had regrown after surgery.

^b^ Signal at 1 week following IV injection confirms successful delivery of SSM3‐Fl cells (experimental metastasis model only).

^c^ Signs of metastasis include luciferase signal during live animal image, ex vivo luciferase signal, visible tumors, and metastasis seen during histological analysis.

^d^ Number refers to mice carried through the experiment beyond the initial injection. One mouse in each group, not counted in table, were euthanised following adverse events that occur immediately after IV injection.

To assess luciferase activity and compare cell line sensitivity, total flux from SSM3‐Fl and SSM3‐A2 derived primary tumors (at a volume of 1000 mm^3^) were recorded over a period of 35–45 min. Signal generated by the SSM3‐Fl primary tumors was greater than the SSM3‐A2 primary tumours with DTZ or NanoGlo substrates (Figure S4). Repeated imaging of SSM3‐A2 tumors was not possible as complete loss of luciferase activity occurred after storage of DTZ for 1 week (Figure S5). While SSM3‐A2 tumors were collected for characterization, all further model development studies were conducted using the SSM3‐Fl cell line.

**FIGURE 2:**
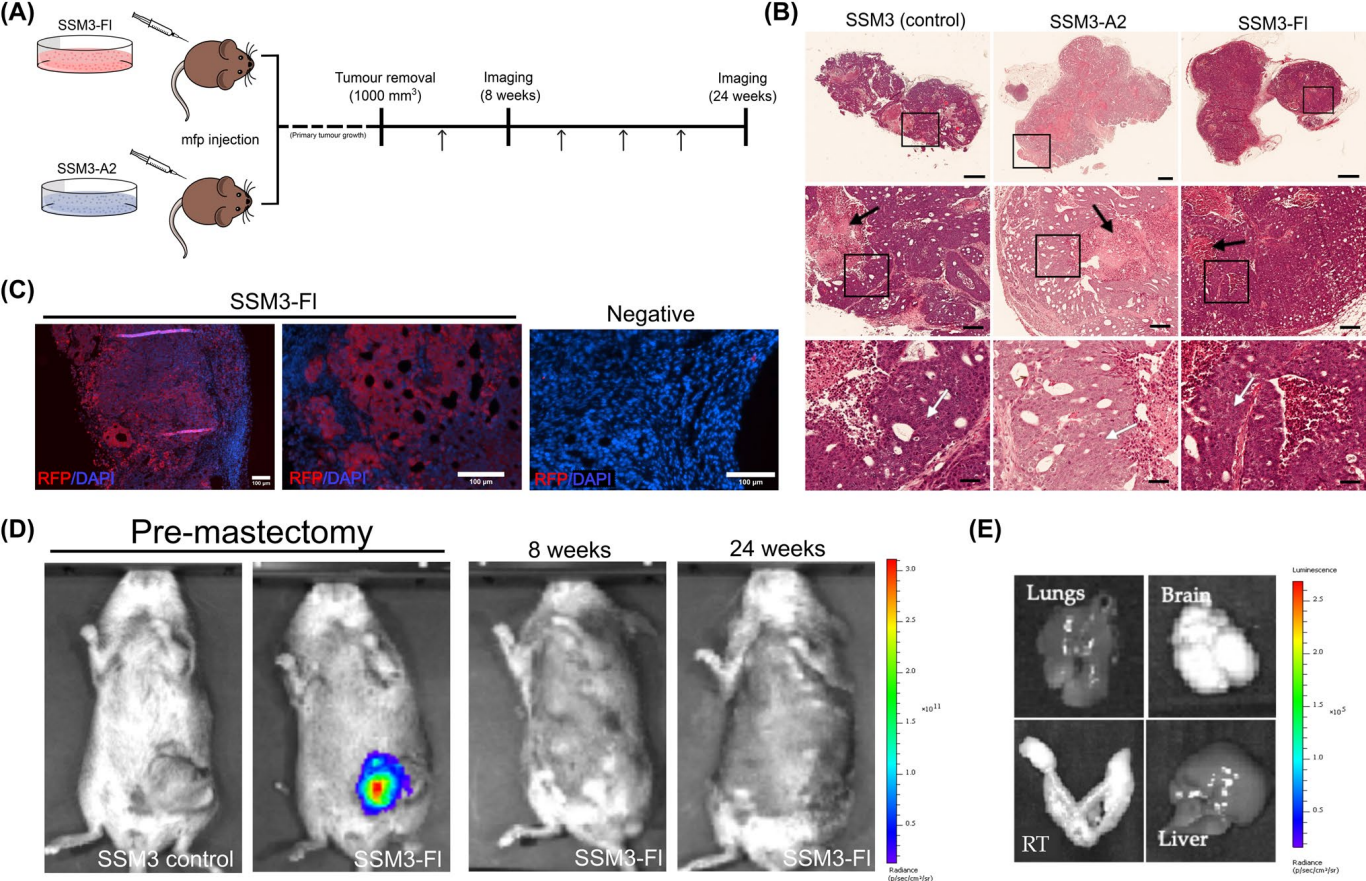
Luciferase tracking of potential metastasis and characterization primary tumor of SSM3‐Fl primary tumors derived from mfp injections in 129S6/SvEv mice. (A) Schematic diagram representing the timeline for the spontaneous metastasis experiments. Mice receiving SSM3‐Fl and SSM3‐A2 cells were directly compared for cell line imaging suitability. Tumors were removed when volume reach 1000 mm^3^ (or before if early signs of ulceration were present). Mice were imaged periodically to monitor disease progression. Representative images displayed from 8‐ and 24‐week time points (arrow indicate additional imaging, not shown). Parental SSM3 cells we also included as imaging controls (not shown). (B) Representative H&E stained sections of SSM3, SSM3‐A2 and SSM3‐Fl primary tumor derived from mammary fat pad injections. Black arrows = areas of necrosis. White arrows = densely populated tumor cells (SSM3 cell lines). Inset boxes correspond to area of magnification. Scale bars; upper panel = 1000 μm, middle panel = 200 μm (5×) and lower panel = 50 μm (20×). (C) Representative IF and DAPI staining of SSM3‐Fl derived primary tumors. This was used to optimize SSM3‐Fl specific IF for future detection of suspected SSM3‐Fl metastasis. A tdTomato/RFP specific primary antibody in conjunction with Alexa Fluor 594 secondary antibody was used to detect SSM3‐Fl cells. Tissue negative control (negative) is from a SSM3‐A2 derived primary tumor stained under the same conditions. Scales bars = 100 μm. (D) Parental SSM3 (SSM3 imaging control) and SSM3‐Fl derived primary tumors were image for luciferase expression when tumors reached 1000 mm^3^ (upper panel). Primary tumors were then removed, and mice were imaged periodically to monitor disease progression for 6 months or unless humane endpoints were reach prior to this. Images of SSM3‐Fl mouse are representative of luciferase activity at 8‐ and 24‐week time points (lower panel). SSM3‐Fl images are matched to the same mouse. Imaging was performed 10 min after IP delivery of 150 mg/kg D‐luciferin and were taken with the IVIS X5 system. Hair on the primary tumor, stomach and chest area as shaved prior to imaging to improve imaging sensitivity. Heat map scale bar displays radiance (photons/s/m^2^/steradian). (E) Ex vivo luciferase imaging of matched lungs, brain, reproductive tract (RT) and liver from SSM3‐Fl mouse. Heat map scale bar = radiance.

Primary tumors were surgically removed when tumors reached a maximum of 1000 mm^3^. Mice were then periodically imaged for 6 months following primary tumor removal surgery (Figure 2A). Tumor regrowth following removal surgery, and/or tumor ulceration, resulted in early termination or early tumor removal, meaning the 6‐month time point was not reached in all mice. Two SSM3‐A2 tumor bearing mice had signs of tumor ulceration resulting in early tumor removal. Regrowth of the primary tumor was seen in 28% and 27% mice with SSM3‐Fl and SSM3‐A2 primary tumors, respectively. Ulceration of tumor regrown after surgery was seen in both mice that received SSM3‐Fl cells respectively (Table 1). Tumors removed during this part of the study were highly cystic, with a large fluid component observed upon removal. This was present in both the luciferase expressing cell lines and the parental SSM3 control. H&E analysis showed that tumors appeared to be made of a fibrotic outer capsule, with central necrosis, as well as cyst‐like structures and blood vessels throughout (Figure 2B). SSM3, SSM3‐A2 and SSM3‐Fl tumors all possessed similar morphology with no notable differences. SSM3 control mice were included as a negative control to validate the specificity of luciferase signal in SSM3‐Fl and SSM3‐A2 primary tumours (Figure 2D).

To confirm that primary tumors were derived from SSM3‐Fl cells, tumors were assessed for tdTomato expression, introduced via the pSBbi‐Luc2 plasmid, using IF. Cells positive for tdTomato can be seen within the mass of the tumor section, with the most intense signal in areas of densely packed tumor cells. Lower levels of fluorescence can be seen in areas of necrosis, in the stromal mantel (tumor capsule), or tumor stroma (Figure 2C).

No negative impacts on the wellbeing and weight of the mice were detected (Figure S6). The seven mice which received SSM3‐Fl mammary fat pad injections had no detectable metastases via live whole animal or ex vivo bioluminescent imaging (Figure 2D,E). Two mice were euthanised prior to 6 months, due to tumor ulceration of the regrown primary tumor (Table 1).

### 3.3 Evaluation of SSM3‐Fl cells for experimentally derived metastasis via intravenous tail injection

**FIGURE 3:**
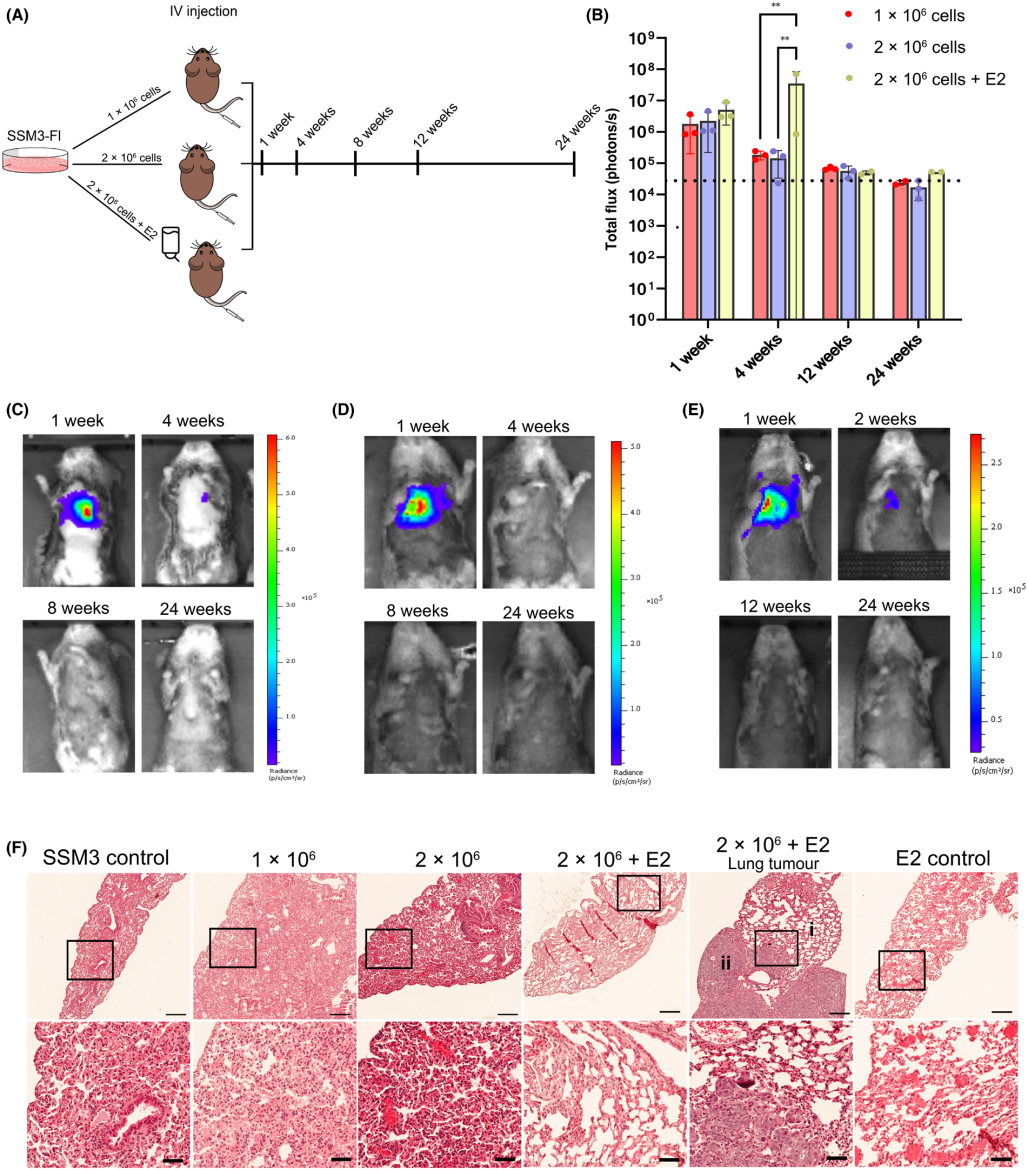
Characterization and luciferase tracking of the experimental metastasis model following delivery of SSM3‐Fl cells through the tail vein of 129S6/SvEv mice. (A) Schematic diagram of experimental metastasis experimental timeline. All mice received SSM3‐Fl cells. (B) Total flux (photons/s) from each mouse was measured at each time point. Bar graph displays average total flux per experimental group at each time point. Circle symbols indicate an individual mouse within each group. Black dashed line indicates the average level of background signal measured during imaging sessions. Error bars indicate standard deviation. Two‐way ANOVA comparing the experimental groups at each time point was carried out, *p* = 0.006 (**). Cell line suspensions in 1X PBS were delivered via IV injection to the lateral tail vein. Mice received either 1 × 10^6^ cells (C), 2 × 10^6^ cells (D) or 2 × 10^6^ cells + E2 supplementation (*n* = 3 per group) (E). Live animal imaging for luciferase expression using the IVIS X5 imaging system was performed at 1, 4, 12 and 24 weeks following cell line deliveries. Imaging was performed 10 min after IP delivery of 150 mg/kg D‐luciferin. Mice stomach/chest area was shaved prior to imaging to reduce the amount of signal blocked due to presence of hair. All time course images are representative and follow one mouse in each group. Heat map scale bars display radiance (photons/s/m^2^/steradian) (C–E). (F) Representative H&E‐stained lung sections from each experimental metastasis group. Upper panel = 5× magnification with 200 μm scale bar. Lower panel = 20× magnification with 50 μm scale bar. 1 × 10^6^ and 2 × 10^6^ (+ E2) panels = number of SSM3‐Fl cells injected IV. i = normal lung tissue, ii = suspected SSM3‐Fl tumor cells. SSM3 control group = 1 × 10^6^ cells delivered IV, E2 control = mouse receiving E2 supplementation only (no cell line injection).

**FIGURE 4:**
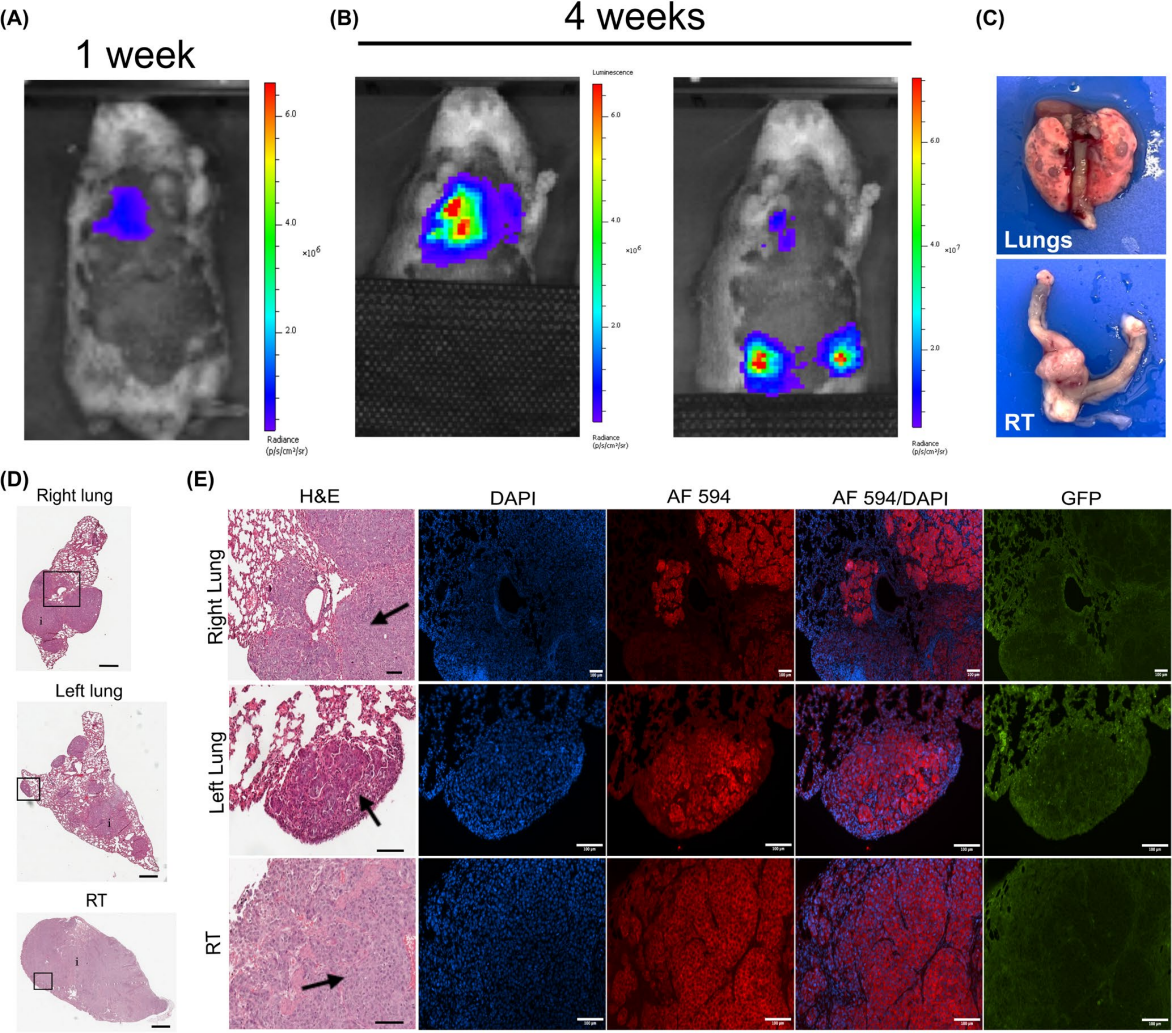
Confirmation of SSM3‐Fl experimental derived metastatic tumors following tail vein injection of 2 × 10^6^ cells and E2 supplementation. (A) Luciferase signal detected in the thoracic cavity of mouse 55092 1 week following IV delivery of SSM3‐Fl cells. (B) Luciferase signal detected in mouse 55092 4 weeks following IV delivery of SSM3‐Fl cells. Left image shows luciferase detected in the thoracic cavity of the mouse, detected through covering more intense signal emitted from the lower abdomen, shown in the image on right. Heat map scale bars (A,B) display radiance (photons/s/m^2^/steradian). (C) Anterior view of lungs and reproductive tract (RT) of mouse 55092 taken at time of necropsy at 8 weeks following IV delivery of SSM3‐Fl cells. (D) H&E of right/left lungs and tumor found on the RT. Scale bar = 500 μm. i = suspected metastatic mass. Inset boxes correspond to areas of magnification shown in E. (E) Matched fluorescent images from suspected metastatic masses found in right/left lungs and RT. Immunofluorescence with tdTomato/RFP specific primary with used with Alexa Fluor 594 secondary antibody to detect SSM3‐Fl cells. Panels show from left to right H&E (black arrows = SSM3‐Fl derived masses), nuclear DAPI stain, tdTomato expression, tdTomato/DAPI merged and GFP (for autofluorescence). Scale bar = 100 μm. All images and histology are from mouse 55092.

To assess whether tumors can be produced experimentally using an IV approach, SSM3‐Fl cells were injected into the tail vein of 129S6/SvEv mice at concentrations of 1 × 10^6^, 2 × 10^6^ or 2 × 10^6^ + E2 supplementation and were monitored for 6 months, unless stated otherwise (Figure 3A, Table 1). Passing cells through a 29G needle had no notable impact on cell viability (Figure S8). Two mice receiving 2 × 10^6^ cells (one from the cell only and one from E2 supplemented groups) where terminated immediately following IV injection of cells due to suspected thromboembolism. Luciferase activity was detected in the thoracic cavity of all experimental groups 1 week after IV delivery of cells (Figure 3B–D). This initial signal detected in mice from the 1 × 10^6^ and 2 × 10^6^ cell groups dropped to levels equivalent to background measurements by 1 month following IV injection (Figure 3B,C,E). In contrast, mice in the 2 × 10^6^ + E2 group had signal that was detectable at 1 month, with one mouse's thoracic luciferase signal increasing (Figures 3D,E and 4A,B). All mice in the 1 × 10^6^ and 2 × 10^6^ groups lacked ex vivo signal in imaged tissue (Figure S7A,B). Two of three mice in the 2 × 10^6^ + E2 group also lacked luciferase activity in ex vivo tissue imaging (Figure S7C). Lungs were fixed in formalin and sectioned for histological analysis. Mice receiving either 1 × 10^6^ SSM3, 1 × 10^6^ or 2 × 10^6^ SSM3‐Fl cells had no notable changes in lung morphology (Figure 3F). One mouse from the 2 × 10^6^ + E2 group had macroscopic growths visible in both lungs upon necropsy (Figure 3F). All other 2 × 10^6^ + E2 mice had no notable changes. The E2 control mouse, used as to monitor long term effects of E2, had no changes in lung morphology (Figure 3F).

### 3.4 Confirmation of IV injection‐ derived SSM3‐Fl metastatic tumors in the 129S6/SvEv mouse background

To confirm that tumors in the mouse described above were derived from the injected SSM3‐Fl cells, additional histopathological analysis was conducted on the tissues from this animal. Luciferase activity had increased in the chest between 1 and 4 weeks, and was also detected in the approximate location of the ovaries (Figure 4A,B). At 8 weeks, following the IV injection, the mouse had a sudden decline in overall health, resulting in early termination. Tumors were seen macroscopically in each lung, ranging from <1 mm to 2 mm (Figure 4C). A single large growth on the outside of the right uterine horn was observed (Figure 4C). Suspected lung tumors were solid masses with boundaries that extended past the margins of the lungs (Figure 4C,D). The cellular arrangement of the uterine mass was like that of the lung masses. The uterine growth extended beyond the uterus with undefined boundaries and no normal uterine tissue could be seen in the sections examined (Figure 4D). Masses were positive for tdTomato by IF, confirming that the tumors were derived from SSM3‐Fl cells (Figure 4E). Staining can be seen throughout the tumors and is absent in other structures such as the tumor stromal mantel, or surrounding tissue in both lung and uterus sections (Figure 4E, Figure S8).

### 3.5 Establishment of the MIND model in the 129S6/SvEv background

**FIGURE 5:**
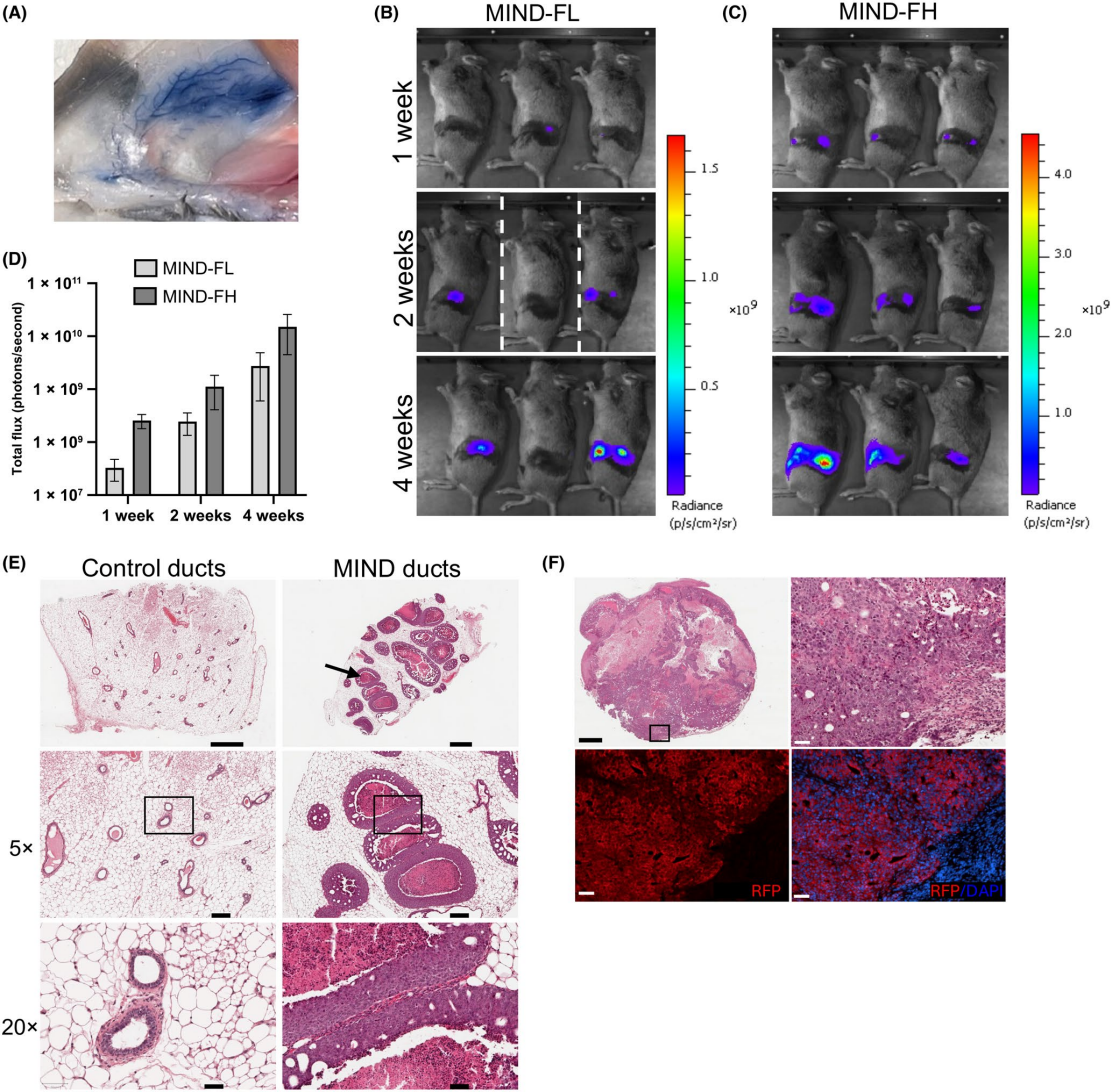
Establishment of the MIND model in the 1296S/SvEv background (A) Mammary ducts Injected with trypan blue dye for visualization of ductal network. MIND injections were then carried out with SSM3 or SSM3‐Fl cells. (B, C) Live luciferase imaging of mice in the low (MIND‐FL, 5 × 10^4^ SSM3‐Fl) or high (MIND‐FH, 1 × 10^5^ SSM3‐FL cell) cell number groups over 4 weeks. Mice are matched in each time point image. Heat map scale bars display radiance (photons/s/m^2^/steradian). (D) Bar graph of total flux detected over 4 weeks from the MIND Injection mammary duct. Error bars show standard deviation. (E) H&E of control (left) and MIND mammary glands (right). Scales bars; upper panel = 1000 μm, middle panel = 200 μm (5×) and lower panel = 50 μm (20×). Black arrows = ducts engorged with SSM3 cells. (F) Matched fluorescent images from invasive SSM3‐Fl MIND tumors to confirm masses were SSM3‐Fl derived. tdTomato/RFP specific primary was used with Alexa Fluor 594 secondary antibody to detect SSM3‐Fl cells. Upper panel = H&E images at 2× (scale bar = 1000 μm) and 20× magnification (scale bare = 50 μm), Lower panel = tdTomato expression (left) and tdTomato/DAPI merged tumor sections at 20× magnification (right), scale bars = 50 μm.

This part of the study aimed to establish and optimize the MIND model in the 129S6/SvEv background to generate an immunocompetent model of ER+ breast cancer that could be used in future therapeutic and metastasis studies. Trypan blue was used to visualize ductal structure in the 129S6/SvEv mice (Figure 5A). Following MIND injection of SSM3‐Fl cells, signal was detected throughout the whole ductal network. This signal increased over 4 weeks, indicating the establishment MIND tumors within the ductal network (Figure 5B–D). Two concentrations of cells were assessed for model optimisation. Firstly, the low concentration groups had 5 × 10^4^ cells of either the parental SSM3 (SSM3‐PL) or SSM3‐Fl cells (SSM3‐FL) delivered to the ducts. In addition, a high cell concentration group of 1 × 10^5^ SSM3‐Fl was tested (SSM3‐FH). Mice in the SSM3‐PL group had a 100% establishment rate of primary tumors. Hence, the higher concentration was not tested for the parental SSM3 cells. Mice in the SSM3‐FL group had an approximate 33% establishment rate, whereas 100% establishment was observed from the SSM3‐FH group (Table S1). The time to palpable tumors was also delayed slightly in the SSM3‐FL groups, whereas SSM3‐Pl and SSM3‐FH mice had palpable tumors from 3 weeks (Table S1). Tumors reached the maximum size of 1000 mm^3^ in 5–7 weeks in the SSM3‐Pl and SSM3‐FH groups.

Histology analysis revealed that the ducts were engorged with SSM3 cells and areas of stromal invasion were also evident (Figure 5E). Large primary tumors collected from mice in the SSM3‐Pl, SSM3‐FL and SSM3‐FH groups had complete invasion of the tumor into surrounding stroma, determined through the lack of stromal and ductal structures present (Figure 5F). Tumors also had areas of necrosis within the centres, as observed in the MFP primary tumors mentioned earlier (Figure 5F). SSM3‐Fl tumors in both groups were confirmed with IF. Areas positive for tdTomato indicate the presence of SSM3‐Fl cells, confirming the origin on the MIND tumors (Figure 5F).

## 4 DISCUSSION

Our research endeavored to investigate the 129S6/SvEv mouse model as a model of ER+ mBC and create a novel immunocompetent ER+ model. To do this we generated luciferase‐expressing SSM3 cells to track cancer progression. SSM3‐A2 consistently showed higher luciferase activity in vitro, indicating high sensitivity, in line with previous reports.^23^, ^25^ However, SSM3‐A2 had low levels of detectable Antares2 activity in vivo which is in contrast to previous studies, and would therefore require further optimisation for continued use.^28^, ^29^, ^30^ The SSM3‐Fl cell line provided consistent detectable luciferase activity, consistent with previous research.^31^, ^32^

While systemic delivery of cancer cells via the tail vein is a widely used model of metastasis, it fails to model several key biological processes involved in metastasis.^33^, ^34^, ^35^ In this study, injections of 1 × 10^6^ and 2 × 10^6^ SSM3‐Fl cells alone was insufficient to form metastatic lesions. It is possible that the low cell density in any one area due to systemic delivery combined with the low establishment rates of the tagged SSM3 cells contributed to lack of lesion formation. However, 2 × 10^6^ cells with the addition of continuous E2 supplementation, resulted in lung and uterine tumors. This is consistent with previous studies stating the addition of E2 supports growth of ER responsive tumors and promotes metastasis in ER cancer models.^36^, ^37^ In the future, collection and primary culture of these tumors could be helpful to establish a more highly metastatic cell line.^38^, ^39^ Adverse reactions to the cell line were observed immediately following tail vein injections in two mice, potentially due to thromboembolisms due to tissue factor (CD142) expressed on the SSM3‐Fl cells.^40^, ^41^ This adverse effect would need to be carefully considered in future IV‐based experiments.^38^, ^42^, ^43^

Variable tumor establishment rates were observed in this study from the models utilizing the SSM3‐A2 and SSM3‐Fl cell lines. A potential increase in immunogenicity as a result of fluorophore expression in the SSM3‐A2 and SSM3‐Fl cells, which has previously been observed, may have contributed to low rates of establishment at distant sites.^44^, ^45^, ^46^, ^47^, ^48^ Fluorescent proteins have been reported to cause increased immunogenicity, with more heightened responses being reported toward RFP (and GFP).^45^, ^46^, ^48^ This increased response has been found to interfere with tumorigenesis due to lymphocyte recruitment, with evident alteration in the tumor microenvironment or environment in which introduced tumor cells are present.^44^, ^47^ It would be beneficial to assess whether the tagged cells used in this study are triggering an immune response by analyzing the TME. This could be achieved through analysis of the immune cell infiltrate in the SSM3‐A2 and SSM3‐Fl tumors. Better understanding of the immune landscape in this model could guide future experimental use.

The low rate of metastases from the SSM3 cells in both the mammary fat pad and tail vein mouse models could also be a result of the low metastatic potential of the cells. More aggressive breast cancer cell lines, such as triple negative breast cancer (TNBC), have previously been used in spontaneous and experimental immunocompetent mouse models of metastasis.^49^, ^50^ In contrast, metastatic ER+ breast cancer models are typically conducted as xenografts in immunocompromised mice.^51^, ^52^ This study has shown that the greatest advantage of the 129S6/SvEv model, the intact immune system, may also be one of the biggest challenges. Working with immunocompetent models adds a layer of complexity which may be why a reliable ER+ metastasis model has not yet been fully established. Both models tested either had no or low incidence of metastasis, potentially due to immune cell recruitment resulting in early clearance of delivered cells. Our findings provide important proof‐of‐concept data that support further investigation, acknowledging that although group sizes were limited, they were sufficient to demonstrate the potential of the models. Future studies incorporating larger cohorts are needed to validate and expand on these initial observations, particularly for the further investigation of the IV lung tumor to primary tumor transplant model.

Direct injection of SSM3 cells to the mammary ducts of 129S6/SvEv mice is potentially the most biologically relevant model of ERα positive breast cancer currently available.^22^ In the MIND‐SSM3 model, tumors reliably develop in the presence of hormones and immune cells, more closely resembling the physiological environment of breast cancer, highlighting our interest in its use for translational research. MIND studies have achieved metastasis to the lungs in syngeneic models of TNBC and in immunodeficient mice with grafted cells.^53^, ^54^ In this study tumors reached endpoint volumes before metastasis developed, suggesting that selecting more metastatic SSM3 cells may improve model development.

This study expands the SSM3 model into new primary and metastatic contexts, offering insights for advanced ER+ breast cancer research. We show that systemic delivery establishes clinically relevant lung tumors for potential transplant studies, while the MIND model provides high engraftment rates in an ER+ immunocompetent setting. Although modeling ER+ mBC remains biologically and technically challenging, these advances provide groundwork for future studies of therapeutic approaches in an immunocompetent ER+ context.

## AUTHOR CONTRIBUTIONS

**Devon M. Bull:** Conceptualization; data curation; formal analysis; investigation; methodology; visualization; writing – original draft; writing – review and editing. **Jody Hazlett:** Investigation; methodology; supervision; writing – review and editing. **Emily Schulpen:** Investigation; methodology; writing – review and editing. **Sophie Tunnicliffe:** Data curation; investigation; writing – review and editing. **Alexander D. McLellan:** Methodology; resources; writing – review and editing. **Anita K. Dunbier:** Conceptualization; funding acquisition; methodology; resources; supervision; writing – review and editing.

## FUNDING INFORMATION

This work was funded by the Otago Medical Research Foundation (New Zealand), Grant/Award Number: CT‐382; and Cancer Research Trust (New Zealand), Grant/Award Number: 2011.

## CONFLICT OF INTEREST STATEMENT

The authors declare that they have no conflicts of interest.

## ETHICS STATEMENT

All work with animals was performed in accordance with University of Otago guidelines for animal research and the New Zealand Animal Welfare Act. All procedures and us of animals was approved by the University of Otago Animal Welfare and Ethics Committee. All mouse experiments were conducted under approved animal use protocols: AUP‐19‐193; AUP‐20‐125; AUP‐22‐116 and AUP23‐14. All personnel involved in this animal research had required training provided by the University of Otago Animal Welfare Office.

## Supporting information


**Data S1.** Plasmid map.
**Figure S1**. Plasmid map of pSBbiGP‐Antares2 used in SSM3‐A2 cloning. This plasmid contains the Antares2 insert used for bioluminescent imaging. Co‐transfections with pCMV(CAT)T7‐SB100 results in all elements between the 5′ and 3′ ITLs being cut from the vector and transposed. This plasmid was developed as part of this research and was used in generating the SSM3‐A2 cell line. Plasmid map was created In SnapGene (United States).
**Data S2**. Proliferation assays.
**Figure S2**. Proliferation assays of the SSM3‐A2 and SSM3‐Fl cell lines. Cell proliferation was measured for 4 days. Cells were stained with DAPI and read using the Cytation5 plate reader. Timepoint displays the average cell count per time point, normalized to Day 0, and plotted as a percentage of Day 0. Error bars display the standard deviation, *n* = 3.
**Data S3**. Whole plate flux assay (lower limits).
**Figure S3**. Lower detection limits of the SSM3‐A2 and SSM3‐Fl cell lines. Whole cell luciferase assays were performed using the IVIS X5 bioimaging system to determine the minimum number of cells detectable in vitro. SSM3‐A2 (a) and SSM3‐Fl (b) cells were imaged 10 min after DTZ and d‐luciferin in vitro substrates were added. Each row is representative of a replicate (*n* = 3). Number to left of well images show cell count per well. Heat map parameters were set to display signal above background.
**Data S4**. Kinetic curves.
**Figure S4**. Kinetic curves of SSM3‐A2 and SSM3‐Fl luciferase expression. Luciferase signal was recorded from 3 min following IP injection of substrates. Mice baring SSM3‐A2 tumors received either 100 μL injections DTZ (0.2 μmol) (a) or NanoGlo (FFz, 0.44 μmol) (b) per mouse. Mice baring SSM3‐Fl tumors received 150 mg/kg d‐luciferin (c). Kinetics represented as flux as a percentage of the maximum recorded total flux.
**Data S5**. Primary tumor comparison.
**Figure S5**. Comparison of SSM3‐Fl and SSM3‐A2 derived primary tumors. Primary tumors derived from cell line injections into the 4th mammary fat pad were imaged at 1000mm^3^ prior to tumor removal surgery. Mice bearing SSM3‐Fl tumors were imaged following administration of d‐luciferin (a), and mice bearing SSM3‐A2 tumors were imaged following administration of NanoGlo (b) or DTZ (c). (c) Imaging of SSM3‐A2 primary tumor Initial (left) and 1 week later (left). Mice were imaged with an open emission filter. Total flux (radiance) from tumors was measured for comparison (d) (SSM3‐Fl *n* = 3, SSM3‐A2 + NanoGlo *n* = 3, SSM3‐A2 + DTZ *n* = 2).
**Data S6**. Weight tracking.
**Figure S6**. Weight monitoring in female 129S6/SvEv mice involved in the spontaneous and experimental metastasis models. Mice in the spontaneous model were monitored and weighed from the time of mammary fat pad injection of SSM3, SSM3‐Fl or SSM3‐A2 cells (a). Mice in the experimental model were monitored and weighed from time of IV injection of either SSM3 (cell control), 1 × 10^6^ SSM3‐Fl cells, 2 × 10^6^ SSM3‐Fl cells or 2 × 10^6^ SSM3‐Fl cells + E2 supplementation (b). E2 control = mouse receiving E2 supplementation only (no IV cell line injection). Mice in both studies were weighed a minimum of twice a week. Weight per week is displayed as the average with error bars indicating standard deviation.
**Data S7**. IV necropsy images.
**Figure S7**. Representative ex vivo luciferase images of experimental metastasis mice at the end of 6‐month time course. All mice received cell line injections through the lateral tail vein. Mice were euthanised and tissues were extracted followed by being soaked in 300 μg/μL d‐luciferin solution for 10 min at room temperature prior to imaging. Scale bars represented as counts, indicating no quantifiable signal was detected from any tissues. (a) Tissues from mouse which received 1 × 10^6^ SSM3‐Fl cells. All imaged tissues labeled on figure. (b) Tissues from moue which received 2 × 10^6^ SSM3‐Fl cells. Wells contains; (1) kidneys, heart, (2) intestines, (3) liver, (4) lungs, (5) brain, spleen, femur, and (6) reproductive tissues. (c) Tissues from mice that received 2 × 10^6^ SSM‐Fl cells + E2 supplementation. Wells contain lungs from; (1) mouse 55 095 and (2) mouse 55 976.
**Data S8**. SSM3 following pass through 29G needle.
**Figure S8**. Cell viability and adherence following being passed through a 29G needle. SSM3 cells were grown in normal culture conditions. (a) Passing cells through a 29G needle did not have an impact on cell viability. Cells were passed through a 29G insulin needle (blue) and immediately assessed (pre‐incubation) for viability or seeded at a density of 50 000 and incubated for 48 h before being assessed for changes in viability (post‐incubation). A 1:1 ratio of cell suspension to 0.4% trypan blue was used to determine viability. Cell suspensions were analyzed on an automatic cell counter (Luna II, Logos Biosystems, South Korea). Control (blue) = SSM3 cell that were lifted and suspended only, no needle pass. Needle (orange) = cells that were passed through a 29G needle. (b) SSM3 cells that passed through a 29G needle had no change in adherence in vitro. Cells were seeded in triplicate at a density of 50 000 cells and incubated for 48 h. Cells were fixed and stained with 1.5 mg/mL Saponin, 0.5% PFA, and 2 μg/mL Hoescht. Cell counts were taken on the Cytation X5. representative square sections were counted for each well. Control (green) = SSM3 cell that were lifted and suspended only, no needle pass. Needle (purple) = cells that were passed through a 29G needle.
**Data S9**. Immunofluorescence controls.
**Figure S9**. Controls for IV metastasis immunofluorescence. All controls were imaged for RFP expression. Positive control = SSM3‐Firefly derived mammary fat pad tumor. Tissue negative control = SSM3‐Antares2 derived primary tumor, control for SSM3 specific autofluorescence. 1° only (lung tumor) = tdTomato primary antibody only. 2° only (lung tumor) = Alexa Fluor 594 secondary antibody only, control for non‐specific binding to lung tumor. No stain = No stan control on control lung tissue to determine lung tissue autofluorescence. 2° only (uterus tumor) = Alexa Fluor 594 secondary antibody only, control for non‐specific binding to uterus tumor (Scale bars = 25 μm). E2 control (lung and uterus) = control tissue from mouse receiving E2 supplementation (no cell line Injection) stained with tdTomato primary antibody and Alexa Fluor 594 secondary antibody. All scale bars = 50 μm (unless stated otherwise).
**Table S1**. Summary of 1296S/SvEv MIND model key data.

## Data Availability

Data generated in this study is available upon reasonable request from the authors.
