# Supplementary material for: Development of immunocompetent models for primary and metastatic ER+ breast cancer
Source: Animal Model Exp Med. 2026 May 12;9(5):866–78. doi: 10.1002/ame2.70210 (PMC13331550; doi:10.1002/ame2.70210)
Supplement: Supplementary file 1 — Data S1. Plasmid map. Figure S1. Plasmid map of pSBbiGP‐Antares2 used in SSM3‐A2 cloning. This plasmid contains the Antares2 insert used for bioluminescent imaging. Co‐transfections with pCMV(CAT)T7‐SB100 results in all elements between the 5′ and 3′ ITLs being cut from the vector and transposed. This plasmid was developed as part of this research and was used in generating the SSM3‐A2 cell line. Plasmid map was created In SnapGene (United States). Data S2. Proliferation assays. Figure S2. Proliferation assays of the SSM3‐A2 and SSM3‐Fl cell lines. Cell proliferation was measured for 4 days. Cells were stained with DAPI and read using the Cytation5 plate reader. Timepoint displays the average cell count per time point, normalized to Day 0, and plotted as a percentage of Day 0. Error bars display the standard deviation, n = 3. Data S3. Whole plate flux assay (lower limits). Figure S3. Lower detection limits of the SSM3‐A2 and SSM3‐Fl cell lines. Whole cell luciferase assays were performed using the IVIS X5 bioimaging system to determine the minimum number of cells detectable in vitro. SSM3‐A2 (a) and SSM3‐Fl (b) cells were imaged 10 min after DTZ and d‐luciferin in vitro substrates were added. Each row is representative of a replicate (n = 3). Number to left of well images show cell count per well. Heat map parameters were set to display signal above background. Data S4. Kinetic curves. Figure S4. Kinetic curves of SSM3‐A2 and SSM3‐Fl luciferase expression. Luciferase signal was recorded from 3 min following IP injection of substrates. Mice baring SSM3‐A2 tumors received either 100 μL injections DTZ (0.2 μmol) (a) or NanoGlo (FFz, 0.44 μmol) (b) per mouse. Mice baring SSM3‐Fl tumors received 150 mg/kg d‐luciferin (c). Kinetics represented as flux as a percentage of the maximum recorded total flux. Data S5. Primary tumor comparison. Figure S5. Comparison of SSM3‐Fl and SSM3‐A2 derived primary tumors. Primary tumors derived from cell line injections into the 4th mammar [file AME2-9-866-s001.docx]

Supplementary 1 – Plasmid map


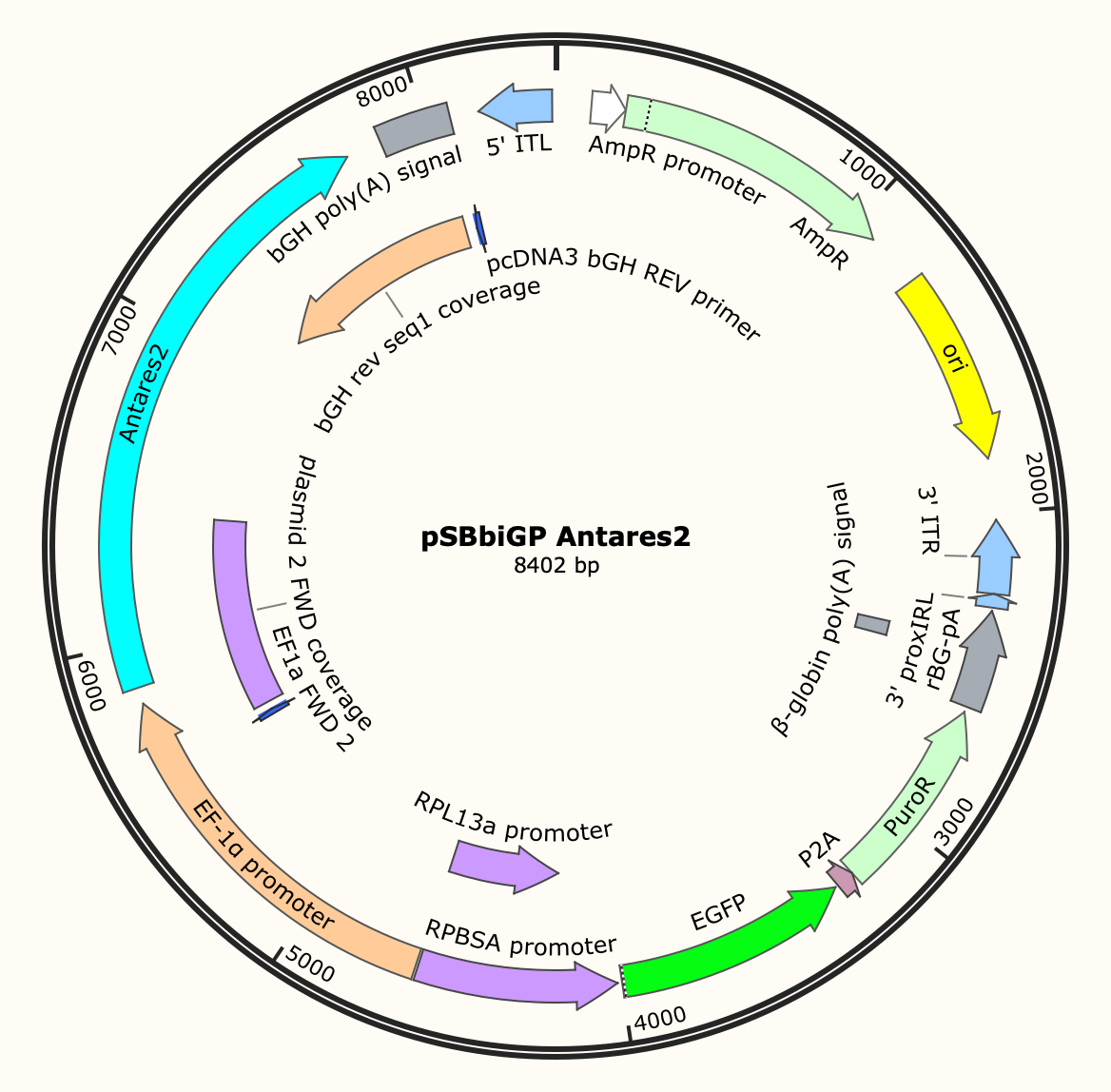


**Figure S1 Plasmid map of pSBbiGP-Antares2 used in SSM3-A2 cloning**. This plasmid contains the Antares2 insert used for bioluminescent imaging. Co-transfections with pCMV(CAT)T7-SB100 results in all elements between the 5’ and 3’ ITLs being cut from the vector and transposed. This plasmid was developed as part of this research and was used in generating the SSM3-A2 cell line. Plasmid map was created In SnapGene (United States).

Supplementary 2 – Proliferation assays


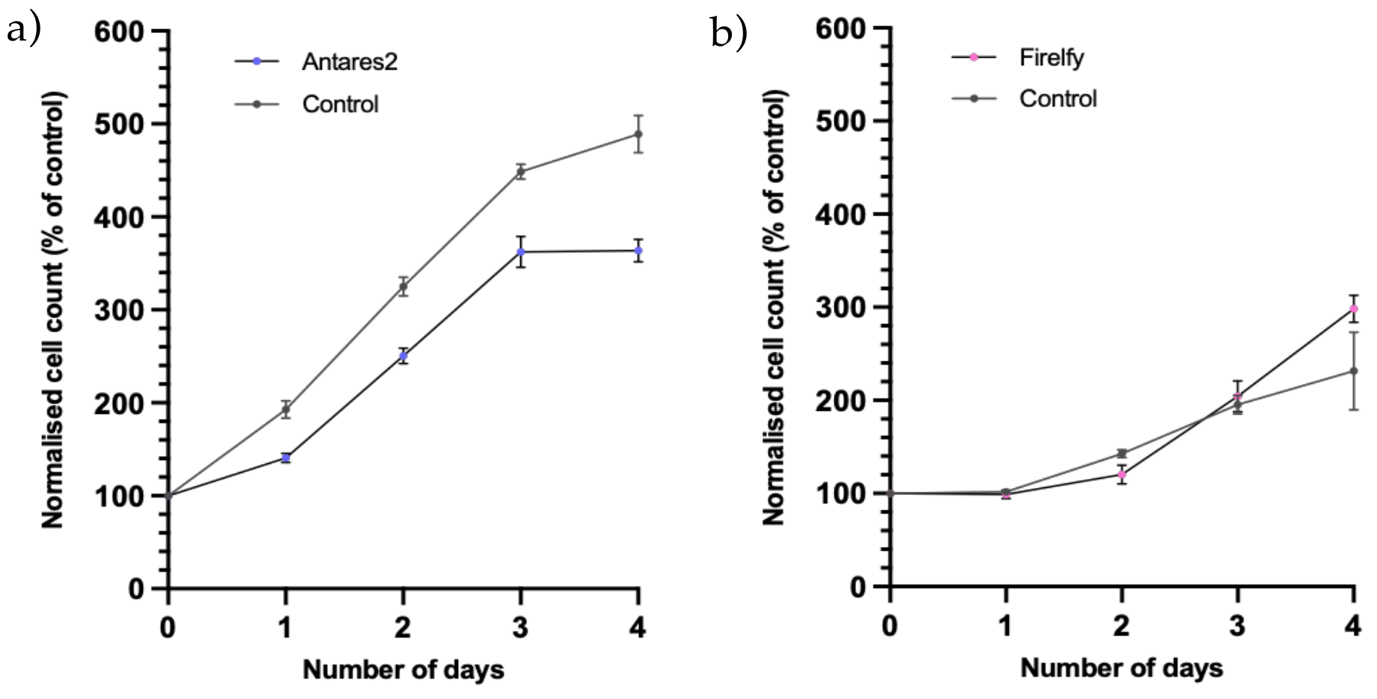


**Figure S2 Proliferation assays of the SSM3-A2 and SSM3-Fl cell lines**. Cell proliferation was measured for four days. Cells were stained with DAPI and read using the Cytation5 plate reader. Timepoint displays the average cell count per time point, normalised to day 0, and plotted as a percentage of day 0. Error bars display the standard deviation, n=3.

Supplementary 3 – Whole plate flux assay (lower limits)


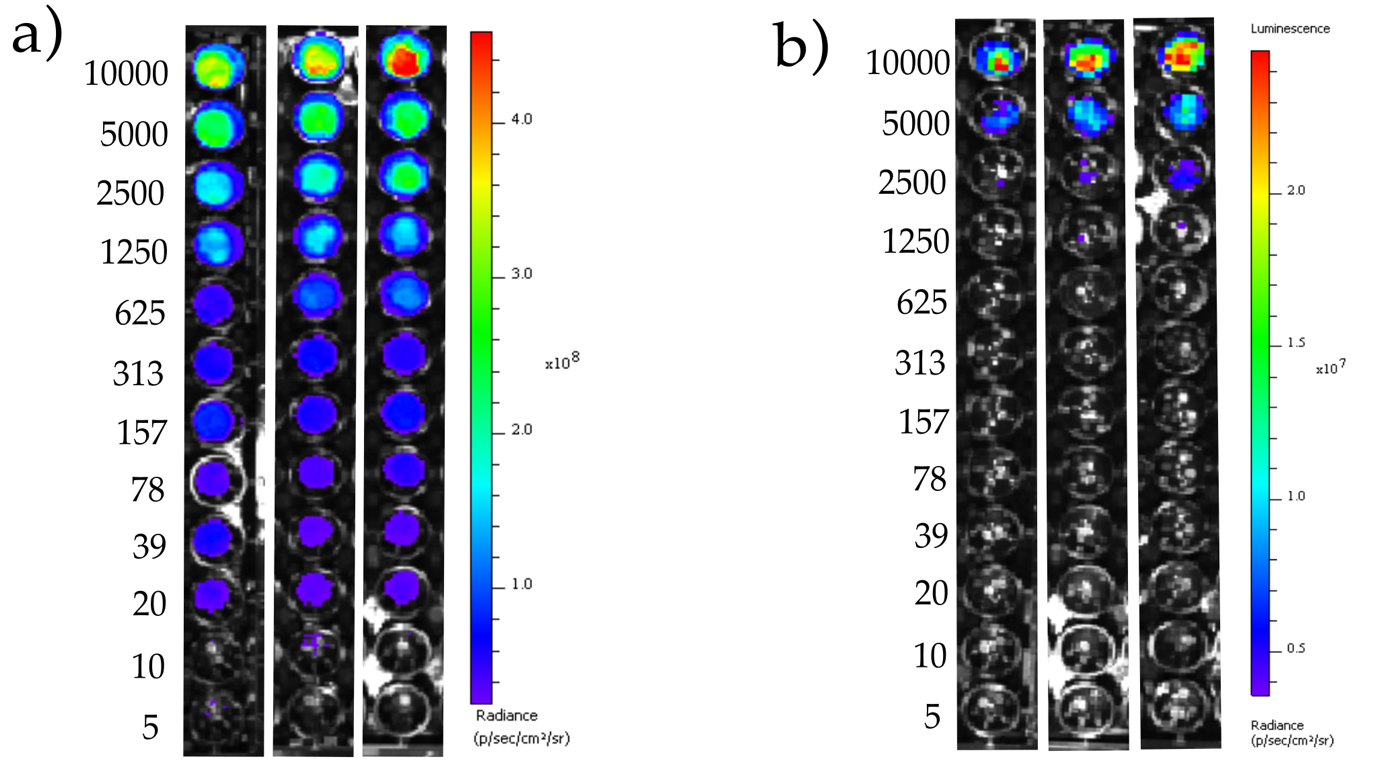


**Figure S3 Lower detection limits of the SSM3-A2 and SSM3-Fl cell lines.** Whole cell luciferase assays were performed using the IVIS X5 bioimaging system to determine the minimum number of cells detectable *in vitro*. SSM3-A2 (a) and SSM3-Fl (b) cells were imaged 10 minutes after DTZ and d-luciferin *in vitro* substrates were added. Each row is representative of a replicate (n=3). Number to left of well images show cell count per well. Heat map parameters were set to display signal above background.

Supplementary 4 – Kinetic curves


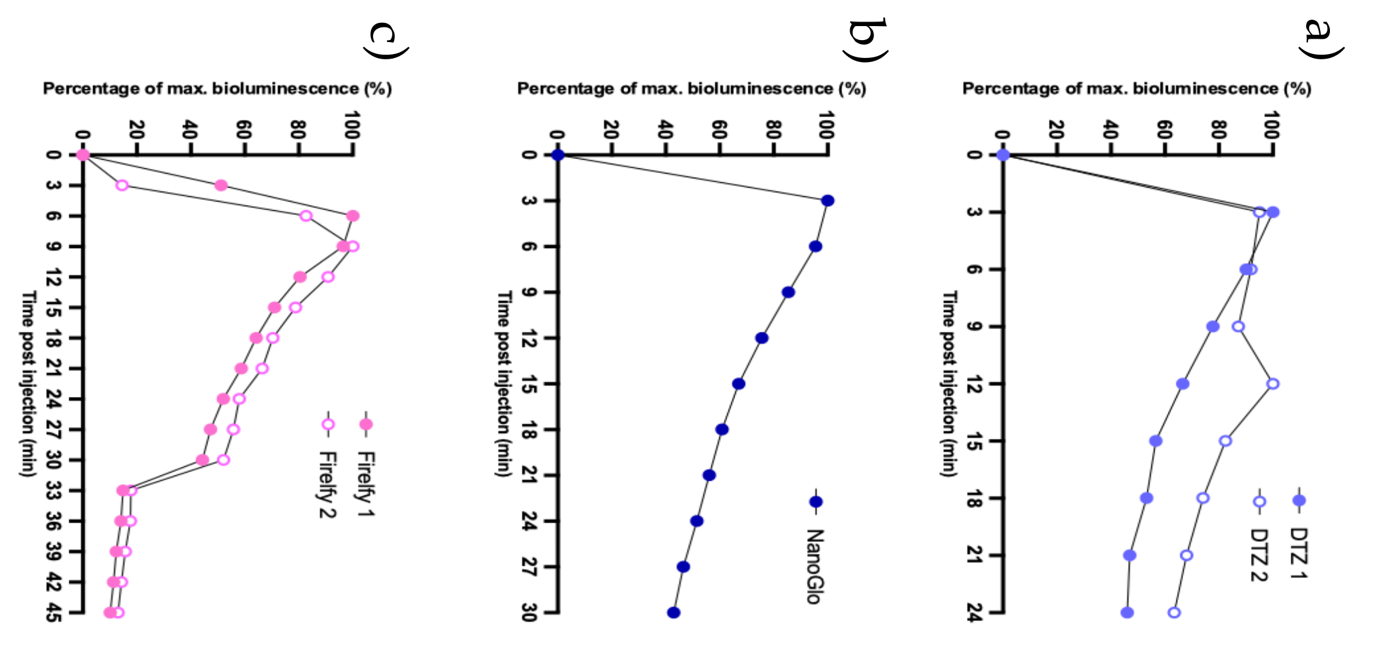


Figure S4 Kinetic curves of SSM3-A2 and SSM3-Fl luciferase expression. Luciferase signal was recorded from 3 minutes following IP injection of substrates. Mice baring SSM3-A2 tumours received either 100 µL injections DTZ (0.2 µmol) (a) or NanoGlo (FFz, 0.44 µmol) (b) per mouse. Mice baring SSM3-Fl tumours received 150 mg/kg d-luciferin (c). Kinetics represented as flux as a percentage of the maximum recorded total flux.


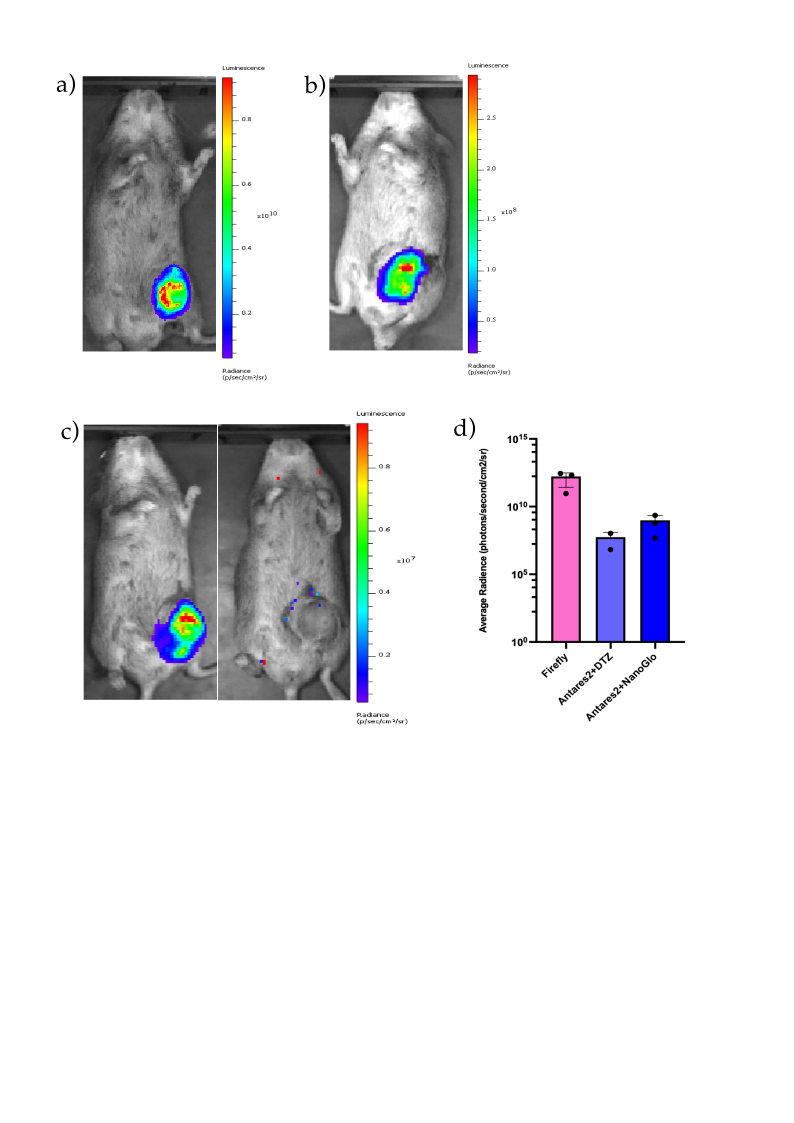
Supplementary 5 – Primary tumour comparison

**Figure S5 Comparison of SSM3-Fl and SSM3-A2 derived primary tumours.** Primary tumours derived from cell line injections into the 4^th^ mammary fat pad were imaged at 1000mm^3^ prior to tumour removal surgery. Mice bearing SSM3-Fl tumours were imaged following administration of d-luciferin (a), and mice bearing SSM3-A2 tumours were imaged following administration of NanoGlo (b) or DTZ (c). c) Imaging of SSM3-A2 primary tumour Initial (left) and one week later (left). Mice were imaged with an open emission filter. Total flux (radiance) from tumours was measured for comparison (d) (SSM3-Fl n=3, SSM3-A2 + NanoGlo n=3, SSM3-A2 + DTZ n=2).


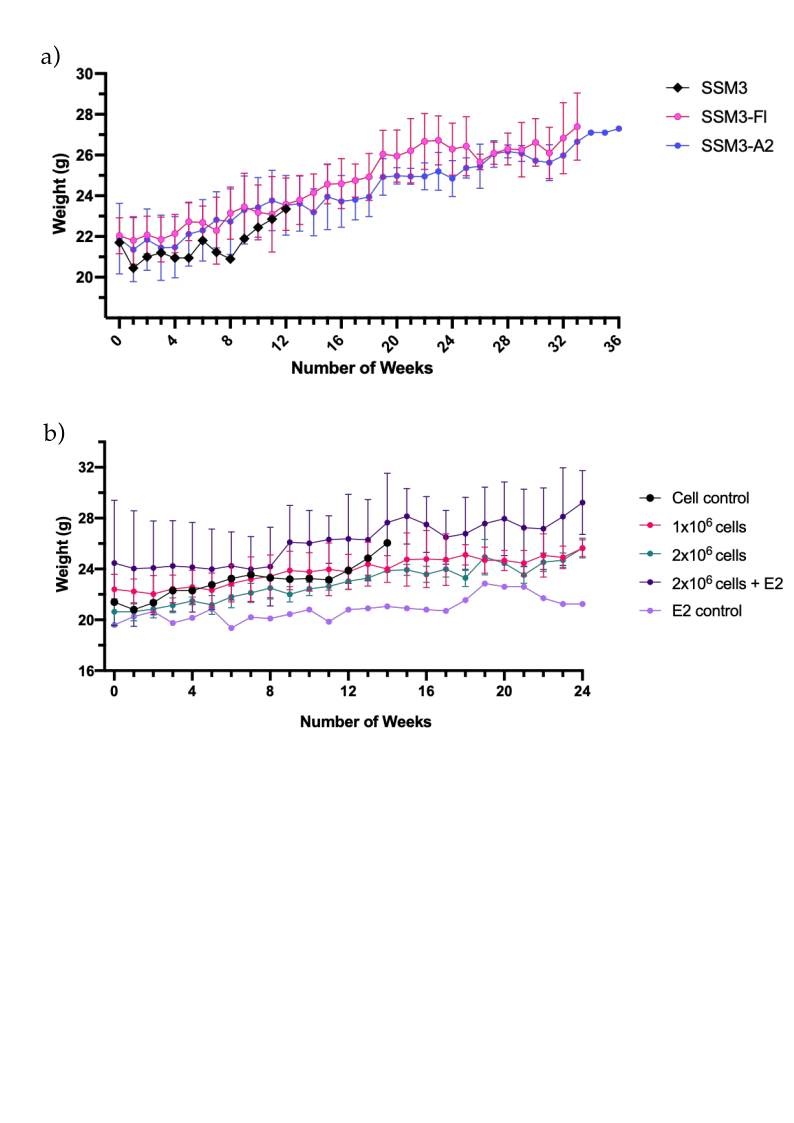
Supplementary 6 – Weight tracking

**Figure S6 Weight monitoring in female 129S6/SvEv mice involved in the spontaneous and experimental metastasis models.** Mice in the spontaneous model were monitored and weighed from the time of mammary fat pad injection of SSM3, SSM3-Fl or SSM3-A2 cells (a). Mice in the experimental model were monitored and weighed from time of IV injection of either SSM3 (cell control), 1x10^6^ SSM3-Fl cells, 2x10^6^ SSM3-Fl cells or 2x10^6^ SSM3-Fl cells + E2 supplementation (b). E2 control = mouse receiving E2 supplementation only (no IV cell line injection). Mice in both studies were weighed a minimum of twice a week. Weight per week is displayed as the average with error bars indicating standard deviation.

Supplementary 7 – IV necropsy images

c)

b)

a)


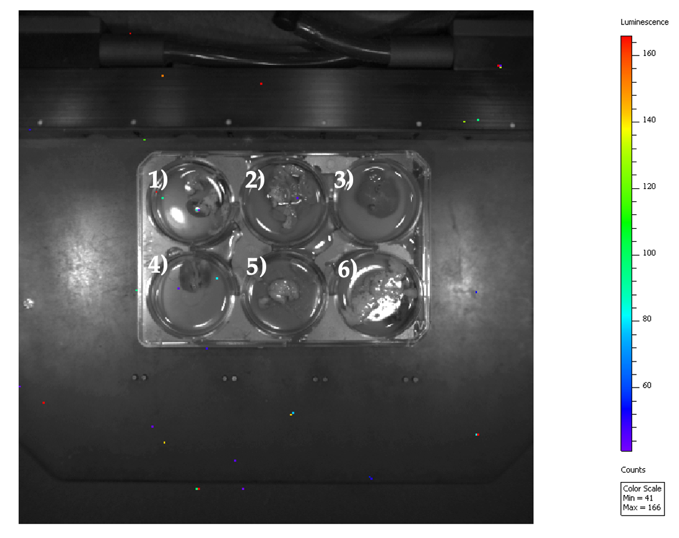

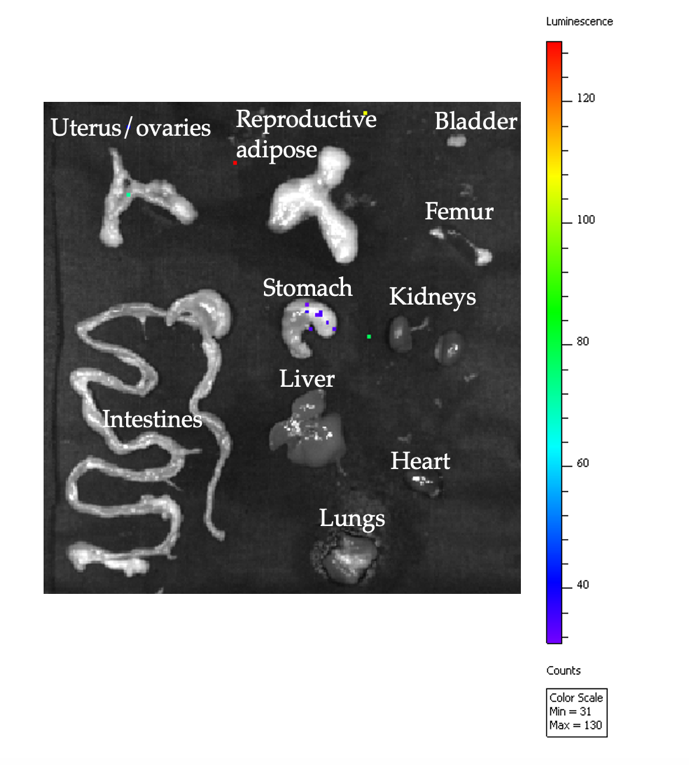

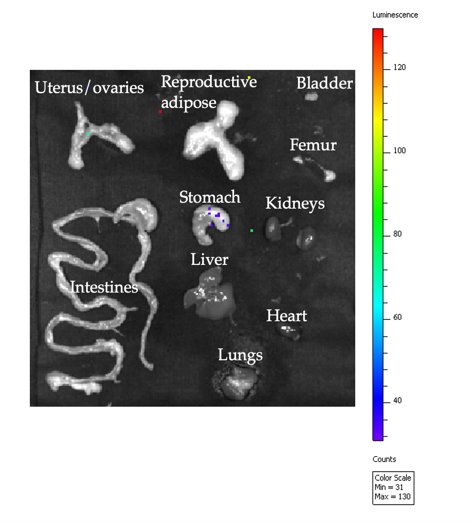

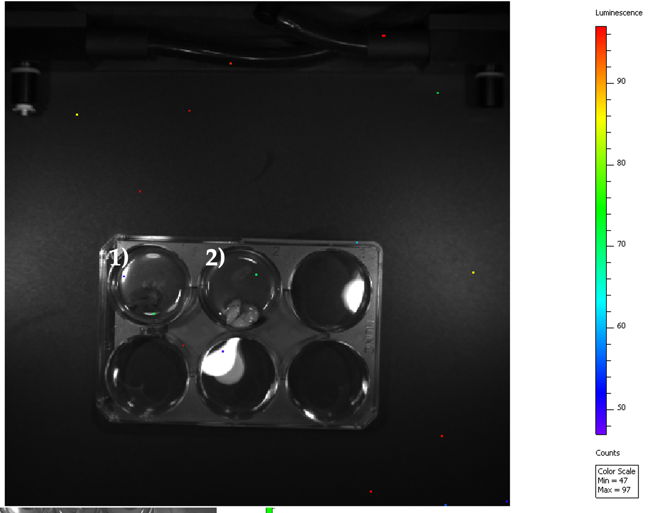


**Figure S7 Representative *ex vivo* luciferase images of experimental metastasis mice at the end of 6-month time course.** All mice received cell line injections through the lateral tail vein. Mice were euthanised and tissues were extracted followed by being soaked in 300 µg/µL d-luciferin solution for 10 minutes at room temperature prior to imaging. Scale bars represented as counts, indicating no quantifiable signal was detected  from any tissues. (a) Tissues from mouse which received 1x10^6 SSM3-Fl cells. All imaged tissues labelled on figure. (b) Tissues from moue which received 2x10^6 SSM3-Fl cells. Wells contains; 1) kidneys, heart, 2) intestines, 3) liver, 4) lungs, 5) brain, spleen, femur, and 6) reproductive tissues. (c) Tissues from mice that received 2x10^6 SSM-Fl cells + E2 supplementation. Wells contain lungs from; 1) mouse 55095 and 2) mouse 55976.

Supplementary 8 - SSM3 following pass through 29G needle


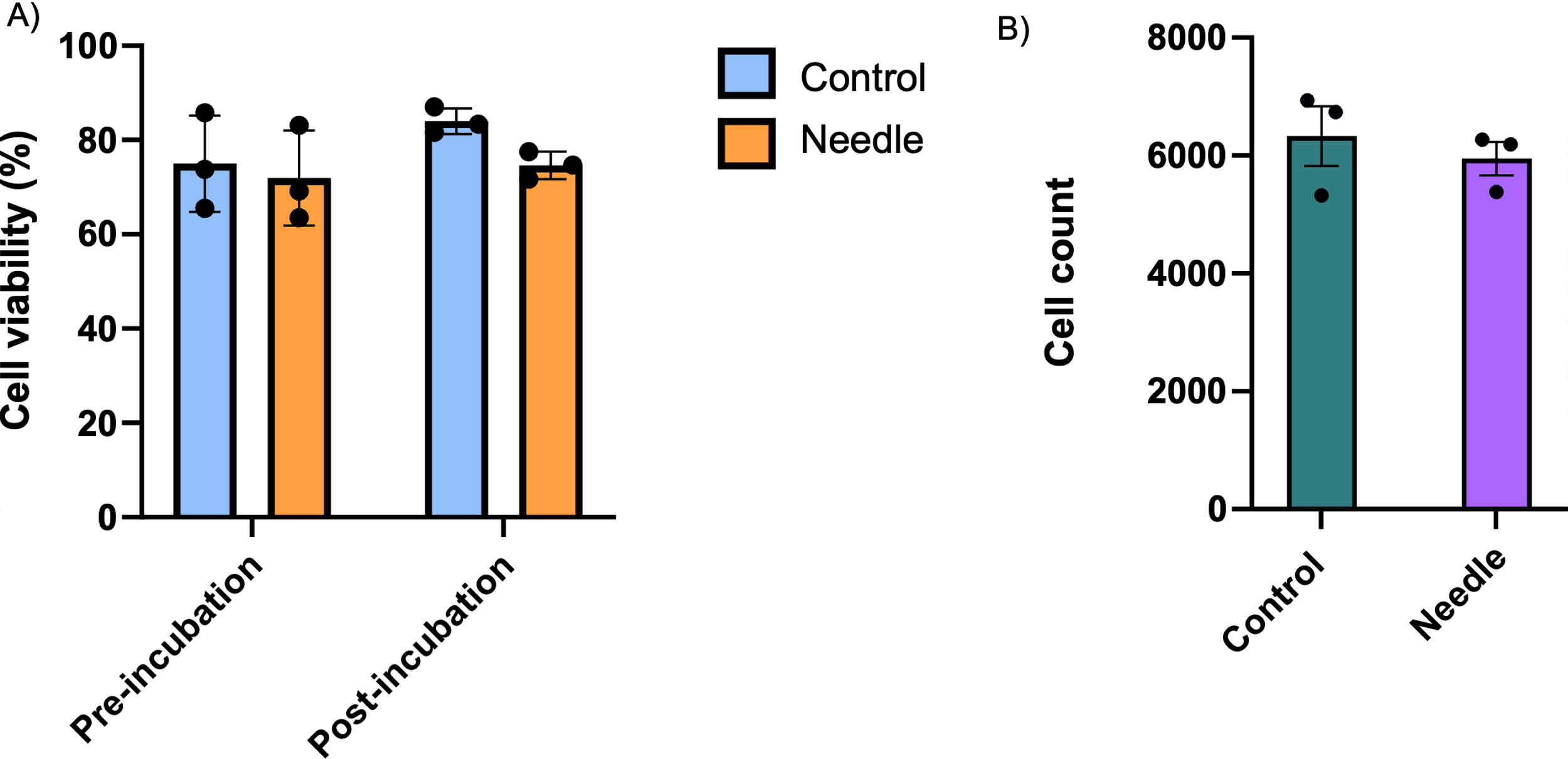


a)

b)

**Figure S8 Cell viability and adherence following being passed through a 29G needle.** SSM3 cells were grown in normal culture conditions. (a) Passing cells through a 29G needle did not have an impact on cell viability. Cells were passed through a 29G insulin needle (blue) and immediately assessed (pre-incubation) for viability or seeded at a density of 50,000 and incubated for 48 hours before being assessed for changes in viability (post-incubation). A 1:1 ratio of cell suspension to 0.4% trypan blue was used to determine viability. Cell suspensions were analysed on an automatic cell counter (Luna II, Logos Biosystems, South Korea). Control (blue) = SSM3 cell that were lifted and suspended only, no needle pass. Needle (orange) = cells that were passed through a 29G needle. (b) SSM3 cells that passed through a 29G needle had no change in adherence *in vitro*. Cells were seeded in triplicate at a density of 50,000 cells and incubated for 48 hours. Cells were fixed and stained with 1.5 mg/mL Saponin, 0.5% PFA, and 2 µg/mL Hoescht. Cell counts were taken on the Cytation X5. representative square sections were counted for each well. Control (green) = SSM3 cell that were lifted and suspended only, no needle pass. Needle (purple) = cells that were passed through a 29G needle.


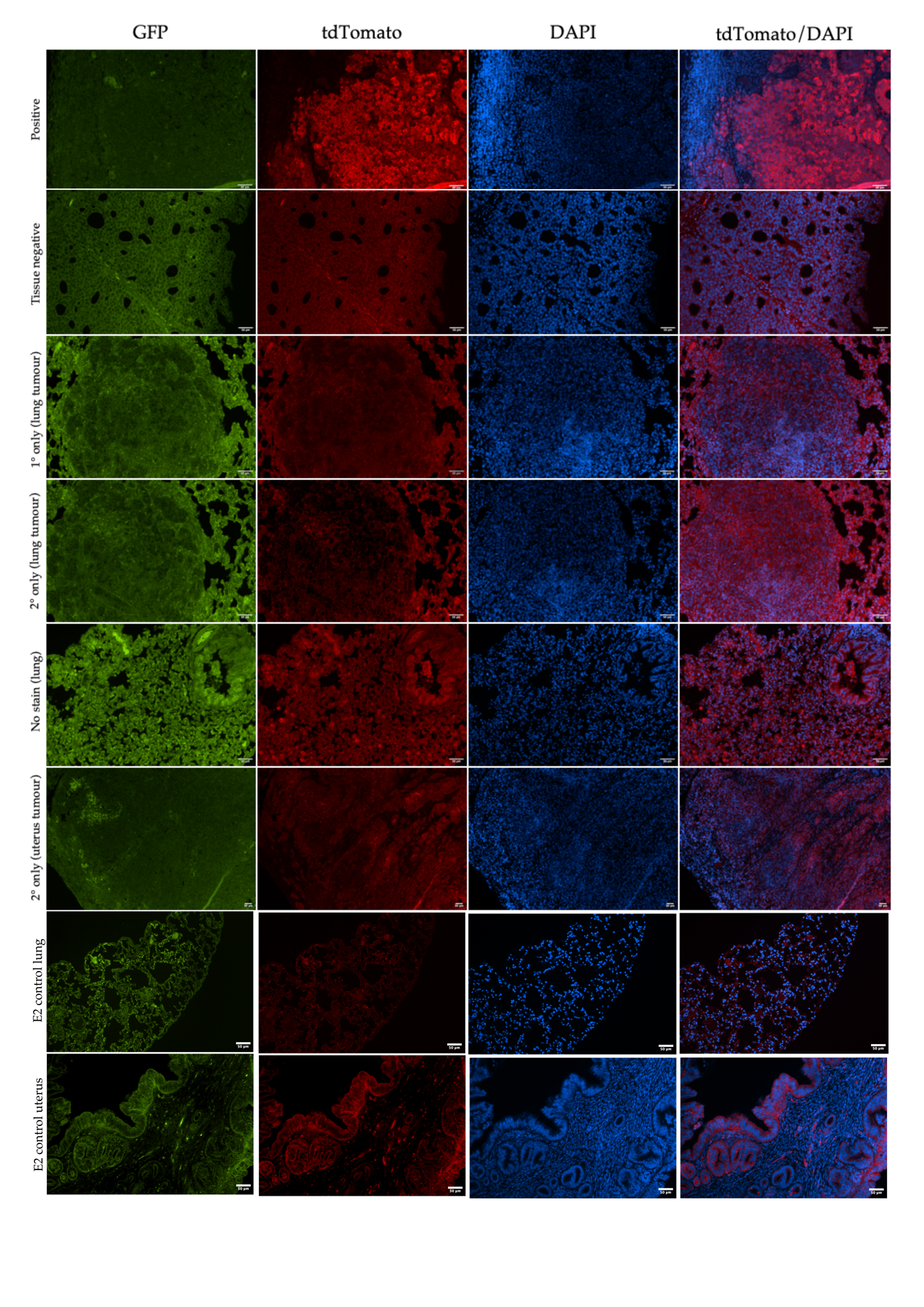
Supplementary 9 - Immunofluorescence controls

Figure S9 Controls for IV metastasis immunofluorescence. All controls were imaged for RFP expression. Positive control = SSM3-Firefly derived mammary fat pad tumour. Tissue negative control = SSM3-Antares2 derived primary tumour, control for SSM3 specific autofluorescence. 1° only (lung tumour) = tdTomato primary antibody only. 2° only (lung tumour) = Alexa Fluor 594 secondary antibody only, control for non-specific binding to lung tumour. No stain = No stan control on control lung tissue to determine lung tissue autofluorescence. 2° only (uterus tumour) = Alexa Fluor 594 secondary antibody only, control for non-specific binding to uterus tumour (Scale bars = 25 µm). E2 control (lung and uterus) = control tissue from mouse receiving E2 supplementation (no cell line Injection) stained with tdTomato primary antibody and Alexa Fluor 594 secondary antibody. All scale bars = 50 µm (unless stated otherwise).

MIND model supplementary table:

| **Table S1: Summary of 1296S/SvEv MIND model key data** | | | | | | |
| --- | --- | --- | --- | --- | --- | --- |
| Experimental group | Number of cells injected | Number of mice included | Time to palpable tumour (weeks) | Number of primary tumours | Time to 1000 mm^3^ (weeks) | Incidence of tumour ulceration |
| MIND-PL | 5x10^4^ | 6 | 3-5 | 6 | 5-6 | 0 |
| MIND-FL | 5x10^4^ | 6 | 6 | 2 | 7 | 0 |
| MIND-FH | 1x10^5^ | 3 | 3-5 | 3 | 5-7 | 0 |
|  |  |  |  |  |  |  |
